# The International Conference on Intelligent Biology and Medicine 2019: computational methods for drug interactions

**DOI:** 10.1186/s12911-020-1051-1

**Published:** 2020-03-18

**Authors:** Xia Ning, Chi Zhang, Kai Wang, Zhongming Zhao, Ewy Mathé

**Affiliations:** 1 Department of Biomedical Informatics, College of Medicine, The Ohio State University, Columbus, OH 43210 USA; 2 Department of Medical & Molecular Genetics, School of Medicine, Indiana University, Indianapolis, Indiana 46202 USA; 3 Raymond G. Perelman Center for Cellular and Molecular Therapeutics, Children’s Hospital of Philadelphia, Philadelphia, PA 19104 USA; 4 Center for Precision Health, School of Biomedical Informatics, The University of Texas Health Science Center at Houston, Houston, TX 77030 USA; 5 Human Genetics Center, School of Public Health, The University of Texas Health Science Center at Houston, Houston, TX 77030 USA

## Abstract

In this editorial, we briefly summarize the International Conference on Intelligent Biology and Medicine 2019 (ICIBM 2019) that was held on June 9–11, 2019 at Columbus, Ohio, USA. Then, we introduce the two research articles included in this supplement issue. These two research articles were selected after careful review of 105 articles that were submitted to the conference, and cover topics on deep learning for drug-target interaction prediction and data mining and visualization of high-order drug-drug interactions.

## Introduction

The International Conference on Intelligent Biology and Medicine 2019 (ICIBM 2019) had four keynote lectures, four eminent scholar talks, five tutorials and workshops, twelve concurrent regular scientific sessions, and one poster session. A total of 164 researchers attended the conference of which 79 were faculty/staff and 84 were trainees. The accepted papers and posters cover a wide range of topics in bioinformatics, next-generation sequencing, single cell analyses, systems biology, intelligent computing, data science, medical informatics and decision making, and others. Among 105 original manuscript submissions, two research articles are of special interest to drug-target interaction and drug-drug interaction prediction. The proposed medical informatics approaches are innovative and have strong translational potentials to facilitate decision making in clinical service. These two articles have gone through peer-review and revision before their final acceptance to this ICIBM2019 supplement issue.

## Summary of selected papers

A key component in drug discovery is to identify protein targets and compounds that are active to the targets. Computational methods play an important role in predicting drug-target interactions (DTIs) due to its high throughput compared to the traditional experimental methods. Wang et al. [1] develop a new model for DTI prediction using deep long short-term memory (DeepLSTM) methods. Their model can extract protein’s evolutionary features from their Position Specific Scoring Matrix (PSSM) and Legendre Moment (LM) and drug molecular substructure fingerprints. Such protein and drug features are mapped into a unified vector space. DeepLSTM is then applied over the protein-drug features to predict their interactions. Their model can achieve an Area under the Curve (AUC) value above 0.92 on four DTI datasets.

Adverse drug events (ADEs) often occur as a result of drug-drug interactions (DDIs). The use of data mining for detecting effects of drug combinations on ADE has attracted growing attention and interest. However, the existing approaches become inefficient from both computational and illustrative perspectives when considering more than three drugs in interactions. Yao et al. [2] develop an efficient approach to estimate the directional effects of high-order DDIs through frequent itemset mining. They also introduce a novel visualization method to organize and present the high-order directional DDI effects involving more than three drugs in an interactive, concise and comprehensive manner. They apply their method to a publicly available FDA Adverse Event Reporting System (FAERS) dataset, confirm previously reported myopathy associated DDIs and uncover a number of novel DDIs leading to increased risk for myopathy.

## Discussion and conclusion

Drug-target interactions and drug-drug interactions provide important medical information that can facilitate decision making in clinical settings when drugs are prescribed. Such research topics emerge at ICIBM 2019 and have drawn signification attention from the conference participants. New methods have been under development particularly via deep learning, large-scale data mining and visualization. We envision a growing interest of medical informatics approaches to address the related prediction problems. We also hope to organize more events similar to ICIBM 2019 to promote computational pharmacology in the areas of medical informatics and machine learning.
