# Correction to: The International Conference on Intelligent Biology and Medicine 2019: computational methods for drug interactions

**DOI:** 10.1186/s12911-020-1096-1

**Published:** 2020-04-28

**Authors:** Xia Ning, Chi Zhang, Kai Wang, Zhongming Zhao, Ewy Mathé

**Affiliations:** 1 Department of Biomedical Informatics, College of Medicine, The Ohio State University, Columbus, OH 43210 USA; 2 Department of Medical & Molecular Genetics, School of Medicine, Indiana University, Indianapolis, Indiana 46202 USA; 3 Raymond G. Perelman Center for Cellular and Molecular Therapeutics, Children’s Hospital of Philadelphia, Philadelphia, PA 19104 USA; 4 Center for Precision Health, School of Biomedical Informatics, The University of Texas Health Science Center at Houston, Houston, TX 77030 USA; 5 Human Genetics Center, School of Public Health, The University of Texas Health Science Center at Houston, Houston, TX 77030 USA

**Correction to: BMC Med Inform Decis Mak. 2020; 20(Suppl 2): 51**

**https://doi.org/10.1186/s12911-020-1051-1**

After publication of this supplement article [1], it is requested the grant ID in the Funding section should be corrected from NSF grant IIS-7811367 to NSF grant IIS-1902617.

Therefore, the correct ‘Funding’ section in this article should read:

We thank the National Science Foundation (NSF grant IIS-1902617) for the financial support of ICIBM 2019. This article has not received sponsorship for publication.
